# A Novel *Clonorchis sinensis* Mitogenome: Elucidating Multiregional Strain Phylogeny and Revising the Digenean Mitochondrial Genome Tree

**DOI:** 10.3390/biom15091246

**Published:** 2025-08-28

**Authors:** Yuxuan Liu, Kaisong Hu, Yanan Zhang, Zhili Chen, Haoyu Zheng, Yuexi Teng, Fang Wang, Jingtong Zheng

**Affiliations:** 1 Department of Pathogen biology, College of Basic Medical Sciences, Jilin University, Changchun 130021, China; yuxuanl24@mails.jlu.edu.cn (Y.L.); yananz23@mails.jlu.edu.cn (Y.Z.); chenzl9922@mails.jlu.edu.cn (Z.C.); zhenghy22@mails.jlu.edu.cn (H.Z.); yxteng25@mails.jlu.edu.cn (Y.T.); 2 Department of Clinical Laboratory, Peking University First Hospital, Beijing 100035, China; 21033@pkufh.com

**Keywords:** *Clonorchis sinensis*, digenean trematodes, mitochondrial genome, multimolecular marker, phylogenetic analysis

## Abstract

*Clonorchis sinensis*, a parasitic liver fluke, is the primary aetiological agent of clonorchiasis, a disease predominantly characterized by liver-related clinical manifestations. Currently, research on the complete mitochondrial (mt) genome of local *C. sinensis* populations remains inadequate. Thus, in this study, we sequenced and annotated the mt genome of fish-borne *C. sinensis* (Cs-c2) from Changchun, Jilin Province, China, a strain not previously described. This mt genome is 14,136 bp in length and harbours 12 protein-coding genes (PCGs), 22 transfer RNAs (tRNAs), 2 ribosomal RNAs (rRNAs), and a single control region (CR). We constructed a maximum likelihood (ML) phylogenetic tree using concatenated *ND5*, *ND6*, and *ND1* from protein-coding genes (PCGs) of the *C. sinensis* mitochondrial genome (mt genome). This tree more clearly differentiated *C. sinensis* strains from three geographical regions (China, Russia, and South Korea) and distinguished Opisthorchiidae from two closely related families (Fasciolidae and Dicrocoeliidae). Additionally, we constructed an ML phylogenetic tree using concatenated *ND4*, *ND5*, *ND1*, *ND2*, and *COX1* from the PCGs of digenean (Digenea) mt genomes. This approach—utilizing multiple high-resolution PCGs with evolutionary rates distinct from those of the mt genome—yielded robust clustering for multiple suborders and 13 families within Digenea and provided new molecular evidence for intergeneric relationships within the suborder Plagiorchiata of Digenea. These findings serve as important references for future research on the differentiation of closely related geographical strains of digeneans, as well as for studies on molecular taxonomy and population genetics.

## 1. Introduction

Tropical diseases, recognized as among the most neglected categories of chronic diseases, pose a significant threat to the health of billions of individuals globally [1]. Digenean flukes constitute a crucial group of parasitic pathogens associated with diverse tropical diseases and exhibit a close aetiological link [2]. Notably, clonorchiasis, caused by *Clonorchis sinensis* parasitizing the intrahepatic bile ducts of humans, is among these diseases. This disease has a global distribution, with a high prevalence, particularly in Southeast Asia, South America, and Africa [3]. Over the past few decades, research on clonorchiasis has advanced considerably in countries such as Japan, South Korea, and China, encompassing multiple domains, such as morphological traits, basic biology, pathogenesis, epidemiology, clinical manifestations, and pharmacotherapeutics [3,4,5,6]. In these areas, elucidating the genetic traits of the pathogen facilitates the analysis of its transmission disparities across different hosts and interspecies genetic variations [7], thereby enabling the tracking of pathogen transmission among diverse hosts as well as its genetic variations within and between host populations. Currently, at certain developmental stages of parasites (e.g., eggs and metacercariae), morphological identification alone fails to accurately differentiate closely related species [8], resulting in frequent misclassification among congeneric species [9]. Phylogenetic analysis offers a novel identification approach for research pertaining to parasite taxonomy and population genetics [10].

In recent years, in addition to advancements in high-throughput sequencing technology [11], the mitochondrial (mt) genome has emerged as a robust tool in molecular biology and genetics [12]. Compared with traditional molecular markers, the mt genome has superior phylogenetic resolution and is thus widely employed as a rapidly evolving molecular marker for inferring phylogenetic relationships among metazoan lineages [13,14]. Unlike ribosomal proteins and other histones (e.g., H1, H2A, H2B, H3, and H4), the mt genome does not form a chromosomal structure [14]. The mt genome primarily undergoes amitosis, ensuring that each daughter cell inherits complete genomic information from the maternal cell (with the maternal origin exhibiting a dihaploid nature) [12]. Owing to the absence of protective proteins such as histones during transmission, the mt genome is highly susceptible to gene mutations, whose evolutionary rate is significantly greater than that of nuclear DNA [15]. Thus, the mt genome—which is characterized by maternal inheritance, a relatively small size, a rapid evolutionary rate, the absence of recombination, a relatively conserved genomic structure, and a growing number of complete mt genome sequences deposited in public databases—serves as a rich source of highly variable genetic markers [10]. The mt genome has been extensively utilized as a genetic marker in research on the taxonomy, phylogenetics, and population genetics of parasitic flatworms [16,17,18].

As a medically important species within the subclass Digenea, *C. sinensis* has been the subject of extensive research, resulting in a considerable body of data on its mitochondrial genome [19]. Wang et al. were the first to complete whole-genome sequencing of the Henan strain from China in 2011, which revealed a circular molecule of 16,258 bp and a tandem repeat region of approximately 4.5 kb, laying the groundwork for subsequent research [20]. Sequencing of the Korean and Russian strains has demonstrated a high degree of conservation in terms of intraspecific genomic structure within this species, along with typical digenean traits [21]. Research on geographical strains has indicated that intraspecific genetic diversity is manifested primarily in length variations in the control region (CR) and base substitutions in certain protein-coding genes (PCGs) [11], with gene sequence similarity exceeding 98% among strains from distinct regions. However, in studies encompassing the entire Asian endemic region, the current geographical scope of research is significantly inadequate, hindering the assessment of genetic differentiation between populations within this region and other geographical strains. This results in insufficient analysis of population genetic structure and cross-regional transmission pathways [22]. Furthermore, within the phylogenetic framework of Digenea, the phylogenetic relationships between Opisthorchiidae (the family to which *C. sinensis* belongs) and related families remain contentious, an issue that cannot be effectively resolved owing to a paucity of molecular-level data [23].

Phylogenetic research on Digenea, one of the most ecologically complex taxa within Platyhelminthes, has advanced despite tensions between morphological convergence and molecular evidence. The core controversies centre on the definition of higher-level taxonomic units and the reconstruction of evolutionary pathways [24]. Traditional classification systems rely on life cycle types (e.g., the presence or absence of third-generation rediae) and morphological traits (e.g., the sucker ratio and arrangement of reproductive organs) [25,26]. However, these characteristics often exhibit convergent evolution under host adaptive pressure [8], resulting in long-standing taxonomic confusion among certain taxa of Plagiorchiida and Echinostomida [27]. Although the application of molecular markers has offered key insights for addressing this issue [12], there remains a paucity of molecular data and definitive bioinformatics approaches to perform adequate phylogenetic analyses and taxonomic interpretations of the subordinate clades within Digenea [28].

Current molecular phylogenetic investigations rely primarily on partial sequences of nuclear ribosomal DNA (rDNA) [29]. Multiple lines of evidence suggest that reliance on a single, relatively conserved nuclear DNA marker may be insufficient to accurately infer relationships among closely related taxa [25,26]. In contrast, mitochondrial genomes—characterized by structural conservation (relatively stable gene composition and arrangement), a moderate evolutionary rate (exhibiting both interspecific discriminability and cross-taxon comparability), and maternal inheritance—have gradually emerged as key molecular markers for resolving higher-level taxonomic controversies within Digenea [10]. However, the application of mitochondrial genomes is not without limitations. For instance, nucleotide composition heterogeneity may induce long-branch attraction bias in phylogenetic trees. Thus, it is necessary to integrate joint analyses of multiple, more resolvable molecular marker genes to calibrate genome-level data, which may facilitate the resolution of genetic relationships among taxonomic groups at all levels within Digenea [30]. In summary, the objectives of this study are as follows: (1) to sequence and annotate the mt genome of a previously unstudied *C. sinensis* strain from Changchun, China (Cs-c2); (2) to conduct phylogenetic analysis among different geographical strains of *C. sinensis* on the basis of the gradient conservation of protein-coding genes in the complete mitochondrial genome, thereby providing support for the structure of its population genetic differentiation; and (3) to perform more extensive phylogenetic analysis on higher taxa of Digenea using the same approach, with the aim of providing more comprehensive molecular evidence for the phylogenetic framework of Digenea.

## 2. Materials and Methods

### 2.1. Parasite Collection and Identification

#### 2.1.1. Collection of Parasitic Materials

Metacercariae were collected in Changchun, Jilin Province. The infected freshwater fish species collected included *Cyprinus carpio* (common carp), *Pseudorasbora parva* (stone moroko), and *Carassius auratus* (crucian carp), in addition to some freshwater shrimps, totalling 200 individuals. Sampling was conducted from September 2022 to March 2025. Parasitized fish and shrimps were obtained from local aquatic markets and fishermen.

Positive individuals were screened for metacercariae using direct compression microscopy. After peeling and chopping, an artificial digestive solution (pepsin digestive solution: 0.7% pepsin, 0.9% NaCl, 0.1% HCl) was added to the minced meat at a ratio of 1:10. The mixture was digested in a 37 °C water bath for 30 min and then filtered through a 200-mesh copper sieve, after which the filtrate was collected. After standing for 30 min, half of the filtrate was collected and washed every 20 min, after which the supernatant was discarded. An appropriate amount of sterile normal saline was added, and this step was repeated until the supernatant became clear. The obtained metacercariae were subjected to morphological identification [31] and then recollected and counted under a microscope. Finally, they were stored at 4 °C until use.

#### 2.1.2. Animals and Experimental Design

Twenty SPF-grade large-eared white rabbits (3 months old, male, weighing 2–2.5 kg) were purchased from Changchun Yisi Laboratory Animal Technology Co., Ltd., (Changchun, China) with the licence number SYXK 2022-0015. The rabbits were housed in a pathogen-free (SPF) environment with a temperature of 22 ± 2 °C, a humidity of 50 ± 10%, and a 12-h light/dark cycle. All protocols involving animal experiments were conducted in accordance with the Guide for the Care and Use of Laboratory Animals of the National Institutes of Health (NIH) and approved by the Animal Experimentation Ethics Committee of Basic Medical College, Jilin University (approval number: 2023-329).

In accordance with previous studies, adult worms were collected by infecting animals with metacercariae [32]. Throughout the experiment, the rabbits had free access to food and water. After 7 days of acclimatization, the rabbits were randomly divided into an infection group (*n* = 10) and a control group (*n* = 10) using a computer-generated random sequence. The inclusion criteria were limited to healthy individuals, and the sample size was determined on the basis of a power analysis from previous experiments [32].

The experiment was performed in a BSL-2 laboratory: the infection group was orally gavaged with a saline suspension containing 80 *C. sinensis* metacercariae (metacercariae isolated from infected fish muscles and verified by microscopic counting), whereas the control group received an equal volume of normal saline (Day 0). The animal status was monitored daily. Eight weeks post-infection, the rabbits were euthanized by intravenous injection of pentobarbital sodium (100 mg/kg) via the ear marginal vein. After dissection, the biliary system was rinsed, and recovered adult worms were counted under a stereomicroscope. The collected adult worms were identified at the species level by morphological observation [31] (Figure S1). Finally, from these qualified adult *C. sinensis* worms, one morphologically typical and structurally intact individual (this isolate was designated as (Cs-c2)) was selected for subsequent genomic DNA extraction and mitochondrial genome sequencing.

### 2.2. DNA Extraction, Long-Range Mt Genome Amplification, and Sequencing

The genomic DNA of *C. sinensis* was extracted using a TIANamp Genomic DNA Kit (TIANGEN, Beijing, China) according to the manufacturer’s instructions. The extracted samples were stored at −20 °C until use. In reference to the published mt genome sequence of *C. sinensis* in GenBank (accession number: FJ381664), four pairs of degenerate primers were designed to target coding regions with high conservation in the genome (*COX1*, *ND1*, *ND4*, and *ND5*) to ensure amplification specificity. These specific primers were synthesized by Sangon Biotech (Shanghai, China) Co., Ltd. (Table S1). We used these four pairs of specific primers to amplify the complete mt genome of *C. sinensis* by employing a series of conventional PCRs. Amplification reactions were performed individually for each pair of primers, and the complete genome was obtained by splicing overlapping fragments.

The PCR system consisted of 10 μL of 2 × M5 HiPer plus Taq HiFi PCR mix (Mei5bio, Beijing, China), 2 μL of template DNA in total (20–50 ng), 0.5 μL each of forwards and reverse primers (25 μM), and ddH_2_O to a final volume of 20 μL. The reaction conditions were as follows: initial denaturation at 95 °C for 3 min; 35 cycles of 94 °C for 25 s, 55 °C for 25 s, and 72 °C for 1 min; and a final extension at 72 °C for 5 min. The PCR products were separated by 1% agarose gel electrophoresis, and the band sizes were observed using an ultraviolet gel imaging system. The target bands were excised, and the products were recovered and purified using a TIANgel Purification Kit (TIANGEN, Beijing, China). The concentration, purity, and integrity of the genomic DNA were subjected to quality control using an Agilent 5400 bioanalyzer (Agilent, Beijing, China). After the DNA samples were qualified, the final DNA solution was sent to Huitong Biotechnology Co., Ltd. (Shenzhen, China) for the construction of next-generation sequencing (NGS) libraries. These library products were subsequently directly subjected to 150 bp paired-end (PE150) high-throughput sequencing on the Illumina HiSeq 2000 platform.

Trimmomatic [33] was used to remove adapter sequences and low-quality reads to reduce bias in the analysis. FastQC v0.11.5 [34] was used to evaluate the quality of the generated sequencing reads. Ultimately, in the *C. sinensis* library, 60,794,984 raw reads and 58,386,602 clean reads were generated (GC content = 44.19%, Q20 = 98.48%, Q30 = 96.70%). The trimmed reads were randomly sampled for subsequent mitochondrial genome assembly.

### 2.3. Mt Genome Sequence Assembly, Annotation, and Analysis

The mitochondrial genome was assembled using a de novo strategy based on Illumina paired-end sequencing data (2 × 150 bp; raw data: 9.12 G; filtered clean data: 8.76 G). SPAdes (v3.14.1) was used for the initial assembly of clean data with default parameters (no cut-off), and contigs were constructed on the basis of the de Bruijn graph (DBG) algorithm and paired-end information. Target sequences were filtered using BLAST v2.16.0 (alignment threshold: ≤1 × 10^−10^) and Exonerate (alignment protein similarity threshold: ≥70%). Combined with the high-copy-number characteristics of mitochondria (average coverage: 1227.36X), GC content, and kmer frequency, fragments whose coverage was significantly lower than that of the main population were removed. The filtered contigs were iteratively extended and merged (50 iterations) using PRICE and MITObim to reduce the number of scaffolds. Subsequently, Bowtie2 [35] was used to map the raw sequencing reads back to the extended sequences; matched paired reads were extracted and reassembled using SPAdes. The above extension-reassembly process was repeated, and the final assembly product was one circular contig with no redundant scaffolds; i.e., a complete mitochondrial genome was confirmed to form a single circular structure through visualization.

The mitochondrial assembly results were aligned with the published complete mt genome of *C. sinensis* (GenBank accession number FJ381664) [11], and genes were annotated using MITOS [36] and MFannot (https://github.com/BFL-lab/MFannot, accessed on 11 March 2024). The genetic codes selected were “Echinoderm and Flatworm Code 9” and “Echinoderm and Flatworm Mitochondrial”. The results of the two annotation methods were integrated, and the start and stop codons of the annotated genes were manually corrected: for transl_table = 9, the start codons were ATG and GTG, and the stop codons were TAA and TAG. The final sequencing and annotation files of the mitochondrial genome sequence of *C. sinensis* obtained in this study were compiled and uploaded to NCBI, with the GenBank accession number NC724724.

The corrected annotated genes were subjected to secondary correction via BLASTnt alignment. MAFFT [37] was used to determine gene boundaries, which were further verified against sequencing results. After confirmation, an mt genome map was generated using CGview [38]. The sequences and secondary structures of transfer RNAs (tRNAs) were predicted using MITOS [36], with their secondary structures visualized and arranged via the RNA 2D Structure (R2DT) tool in RNAiFold for clarity (Figure S2). Additionally, the Visualise R2DT tool in RNAiFold [39] was used to construct rRNA secondary structures (Figure S3). BioEdit 7.0.9 was used to calculate the nucleotide and amino acid compositions and AT/GC skew (Table S2). Finally, in PhyloSuite [40], the annotation files were processed to generate NCBI submission files (.spn) and mt genome statistical tables. The nucleotide sequences of the 12 PCGs were translated into amino acids, and the codon usage and relative synonymous codon usage (RSCU) of the PCGs were calculated; stacked bar charts of the RSCU were plotted using the ggplot2 package in R (4.4.1). The codon usage and RSCU of the *C. sinensis* mt genome from Changchun (Cs-c2) are presented in Figure S4.

### 2.4. Sliding Window Analysis and Synonymous/Nonsynonymous Substitutions

The mt genome sequence of *C. sinensis* (Cs-c2) from the Changchun isolate, China (GenBank accession number: NC724724), and five full-length mt genome sequences of *C. sinensis* from different regions published in GenBank, including South Korean strains (MT607652, KY564177, JF729304), a Russian strain (FJ381664), and a Chinese strain (JF729303), were designated Dataset 1. Basic structural differences among sequences in Dataset 1 were compared.

Coding regions were aligned using the Clustal W v2.1 program built into MEGA-X [41], and sliding window analysis (window size: 100 bp; step size: 20 bp) was performed in DnaSP 6 [42] to calculate nucleotide diversity (π) across regions of the *C. sinensis* mt genome. Additionally, the Changchun isolate of *C. sinensis* (Cs-c2) and PCGs from 30 other digenean trematode mt genomes retrieved from NCBI were designated Dataset 2, and sequence differences in 12 PCGs of Dataset 2 were compared. After alignment by Clustal W, the nonsynonymous/synonymous mutation ratio (Ka/Ks) for the 12 PCGs was calculated using DNASP 6. These calculations were used to roughly assess the relative evolutionary rates, gene selection pressures, and adaptive evolution of PCGs in *C. sinensis* and other digenean trematodes. 

### 2.5. Phylogenetic Resolution and Concatenated Phylogenetic Analysis

On the basis of the mt genome sequencing results, in this study, a discrimination strategy was adopted that prioritizes fast-evolving genes, supplemented by genes with other evolutionary rates, across different taxonomic ranks of Digenea. This strategy utilizes protein-coding genes (PCGs) with high phylogenetic resolution spanning a range of evolutionary rates. Maximum likelihood (ML) was employed for phylogenetic analyses across various taxonomic levels within Digenea. A total of 4 independent datasets were constructed and analysed:

Dataset 1′: Nucleotide sequences of 12 PCGs from the mt genomes of 6 *C. sinensis* strains and some closely related species. Single-gene ML phylogenetic trees were constructed to evaluate the phylogenetic resolving power of individual genes in distinguishing intraspecific relationships among different *C. sinensis* strains and in differentiating multiple closely related families (Figure S5).

Dataset 2′: Nucleotide sequences of 12 PCGs from the mt genome of the *C. sinensis* Changchun isolate (Cs-c2) and 30 other digenean species. Single-gene ML phylogenetic trees were constructed to assess the phylogenetic resolving power of individual genes at higher taxonomic ranks (family/suborder) within Digenea (Figure S6).

Dataset 3: Concatenated nucleotide sequences of 3 PCGs (*ND5*, *ND6*, and *ND1*) from *C. sinensis* strains of different regions and some closely related species. The aim of this dataset is to provide robust phylogenetic inferences for the intraspecific relationships of *C. sinensis* and relationships among closely related species by integrating the combined signals of PCGs with high phylogenetic resolution, which were screened on the basis of the analysis results of Dataset 1′.

Dataset 4: Concatenated nucleotide sequences of 5 PCGs (*ND4*, *ND5*, *ND1*, *ND2*, and *COX1*) from the newly sequenced *C. sinensis* in this study and 30 digenean species retrieved from NCBI. On the basis of the preliminary results of the ML trees constructed from Dataset 2′, these genes were predicted to have high resolution at the family/suborder level; thus, this dataset was used to resolve higher-rank phylogenetic relationships within a broader group of digeneans.

Nucleotide sequences of the PCGs included in all the above datasets were retrieved from GenBank using PhyloSuite, generating .fasta files. Sequence alignment for all datasets was performed directly on nucleotide sequences using the Clustal W program integrated in MEGA-X. Subsequently, Gblocks 0.91b [43] (with strict default parameters) was used to remove ambiguous regions from all alignment results. The optimal nucleotide evolution model was selected on the basis of predictions from the “Find Best DNA/Protein Models” program in MEGA. All the ML phylogenetic trees were evaluated for node support using 1000 bootstrap replicates. The final phylogenetic trees were visualized and annotated using iTOL v7.2.1 [44] and Adobe Illustrator^®^. The predicted results of the optimal evolutionary models for the above datasets are provided in the Supplementary Materials (Table S3).

## 3. Results and Discussion

### 3.1. Basic Characteristics of the Mt Genome of C. sinensis from Changchun Area

**Figure 1:**
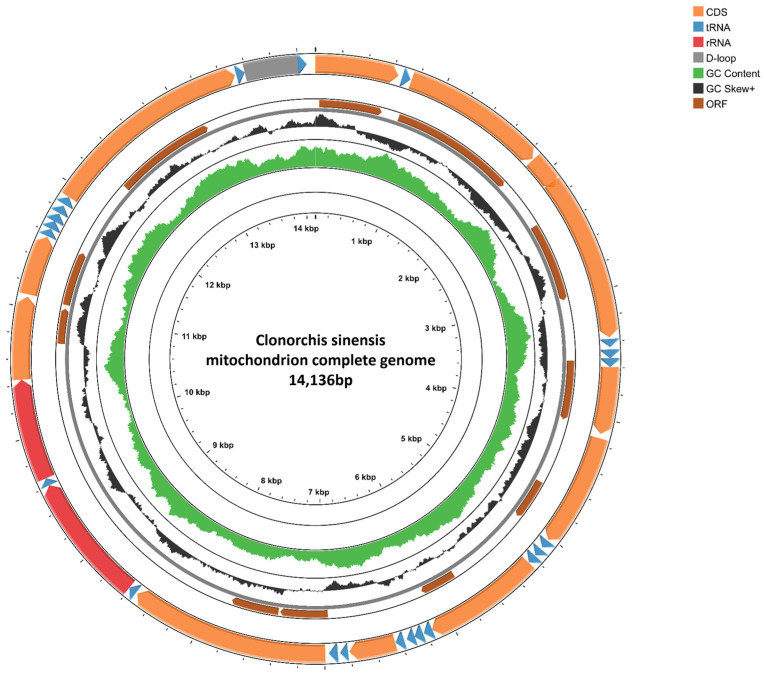
Schematic diagram of the full-length mt genome sequence of *C. sinensis*. The orange, blue, red, grey, and brown bars represent various components of the mt genome. The arrows indicate the transcription direction of each component. The green track represents the G + C content, and the black track represents the GC skew.

The complete mt genome of *C. sinensis* obtained via Illumina high-throughput sequencing in this study has been demonstrated to be sufficient for phylogenetic reconstruction and various comparative analyses [23]. Sequencing revealed that the mt genome of *C. sinensis* from Changchun (Cs-c2) is a typical closed double-stranded circular DNA consisting of 12 protein-coding genes (PCGs), 22 tRNAs, and 2 rRNA genes. A total of 36 genes and two noncoding regions were distributed on the major strand (J-strand), with all the genes transcribed in the same direction (Figure 1). Its annotation characteristics are consistent with the conservative features of the *C. sinensis* mitochondrial genome (Table S4).

Additionally, a 412-bp control region (CR), located between *trnE* and *trnG*, contains gaps and is also known as the D-loop region. The CR, as part of the noncoding regions in the mt genome, plays important roles in replication and transcription regulation [45]. Studies have demonstrated that, as a liver fluke of the Opisthorchiidae family, *C. sinensis* has a gene composition (including PCGs, tRNAs, and rRNAs) and gene order of its mitochondrial genome that are highly consistent with those of the congeneric species *Opisthorchis felineus* and *Opisthorchis viverrini* (within Opisthorchiidae), *Fasciola hepatica* (Fasciolidae), and *Paragonimus westermani* (Paragonimidae). However, interspecific and interfamily differences exist in their nucleotide sequences [46].

### 3.2. Sequencing of the C. sinensis Mt Genome and Comparison of Phylogenetic Relationships

The morphological characteristics of adult worms and metacercariae collected from Changchun, China, were consistent with those described in the literature for *C. sinensis* [47]. The length of the mt genome from the selected single adult worm individual, Cs-c2, is 14,136 bp. A similarity search using the BLAST tool in NCBI revealed that the sequencing results exhibited 99.88%, 99.76%, 99.74%, 99.68%, and 99.60% identity with the full-length mt genome sequences of five *C. sinensis* strains from different regions published in GenBank (Table S5).

Notably, no mt genome sequence of *C. sinensis* from Changchun, China, was deposited in GenBank prior to this study. Among the complete mt genomes of *C. sinensis* uploaded to the NCBI, the Chinese Cs-c1 strain (13,879 bp; JF729303) sequenced by the Gansu Institute of Veterinary Medicine, Chinese Academy of Agricultural Sciences, exhibited only 99.45% identity with the South Korean Cs-k2 strain (13,877 bp; KY564177). The same authors also sequenced the South Korean Cs-k1 strain (13,877 bp; JF729304), with Cs-c1 exhibiting 99.48% identity to Cs-k1, while the two South Korean isolates shared 99.71% identity. This finding indicates that minor variations in mt genome sequences of the same species can be explained by the large geographical distance between sampling locations [23]. Similarly, the clustering of the same species from different regions reflects the phylogenetic resolution of the genes used for tree construction. On this basis, we investigated molecular markers to verify the intraspecific differentiation of *C. sinensis*.

Additionally, ML analysis of the phylogenetic relationships among 31 species from 13 families within Digenea was performed using five screened PCGs. Although the results were useful for revealing interspecific differentiation and relationships among common species in Digenea [48], more precise sequence data and denser taxonomic and geographical sampling are clearly needed to draw definitive conclusions.

### 3.3. Nucleotide Diversity and Nonsynonymous/Synonymous Ratios

**Figure 2:**
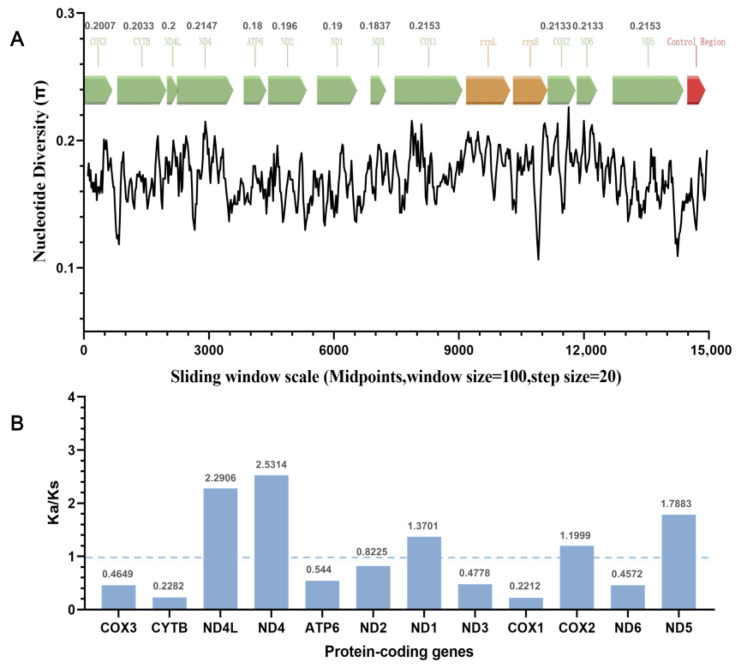
Analysis of the mt genome from Dataset 1 and Dataset 2. (**A**): Sliding window analysis of nucleotides in 12 protein-coding genes (PCGs), 2 rRNAs, and 22 tRNAs from six *C. sinensis* strains. The black line represents nucleotide variation within 100 bp windows (step size = 20 bp, values inserted at midpoints). The gene boundaries of the PCGs, rRNAs, and control region (CR) are distinguished by different colour blocks; the values above each PCG denote the average variation rate of the gene. (**B**): Nonsynonymous/synonymous mutation ratio (Ka/Ks) for 12 PCGs in the mt genomes of 31 trematode species from 13 families within Digenea. Values are labelled above the corresponding coloured bars for each gene.

Previous studies have demonstrated that genes with low π values are often under strong purifying selection or recent selective sweeps and exhibit slow evolution, whereas genes with high π values are primarily under neutral evolution or balancing selection and thus evolve faster. After the mt genome sequences of the Cs-c2 and five other *C. sinensis* strains from different regions were aligned, sliding window analysis (window size = 100; step size = 20) revealed that genes with relatively high intraspecific variability in *C. sinensis* include *COX1* (0.2153), *ND5* (0.2153), *ND4* (0.2147), *ND6* (0.2133), and *COX2* (0.2133); moderately variable genes include *CYTB* (0.2033), *COX3* (0.2007), and *ND4L* (0.2); and *ND2* (0.196), *ND1* (0.19), *ND3* (0.1837), and *ATP6* (0.18) were relatively conserved (Figure 2A).

Calculations of Ka/Ks for 12 PCGs from the Cs-c2 and 30 other digenean trematodes revealed that *ND4* (2.5314), *ND4L* (2.2906), *ND5* (1.7883), *ND1* (1.3701), and *COX2* (1.1999) were under positive selection, driving rapid evolution with increasing adaptive mutation, indicating high evolutionary rates in variable regions. *ND2* (0.8225) and *ATP6* (0.544) exhibit moderate evolutionary characteristics, with their evolutionary rates at an intermediate level, reflecting the balance between gene functional constraints and neutral drift; *ND3* (0.4778), *COX3* (0.4649), *ND6* (0.4572), *CYTB* (0.2882), and *COX1* (0.2212) were under purifying selection, leading to slow evolution and conserved structures (Figure 2B). Previous studies have reported similar high nucleotide variations in *ND4*, *ND5*, and *ND1* of trematode mt genomes [14,17], which was confirmed by Ka/Ks analysis.

Although rRNA genes are commonly used as molecular markers for species identification (“species barcodes”) [30], the accumulation of variations in their sequences, especially in the core functional regions among populations of the same species, is limited. This makes rRNA genes perform stably in phylogenetic analyses at various taxonomic ranks, effectively supporting the inference of genetic relationships between taxa [49]. However, in the differentiation of intraspecific groups (such as different geographical strains of the same species) rRNA genes provide relatively limited discriminatory information because of the low degree of sequence variation [25,26]. Studies have demonstrated that genes with high mutation rates are particularly well suited for resolving recently diverged lineages [50]. Thus, we propose that the use of multiple PCGs, which complement one another in capturing genetic variation, may represent a more effective analytical strategy for identifying *C. sinensis* and revealing phylogenetic relationships among other digenean fluke species [14]. Owing to their ability to capture small-scale genetic variations in helminths, mitochondrial and nuclear gene markers function as high-resolution molecular markers for investigating relationships among closely related organisms [51]. These findings further suggest that distinct regions of the mt genome differ in genetic polymorphism and evolutionary rates across geographically distinct fluke populations, suggesting potential functional divergence among genes [52,53].

### 3.4. Phylogeny of C. sinensis and Other Digenean Trematodes

**Figure 3:**
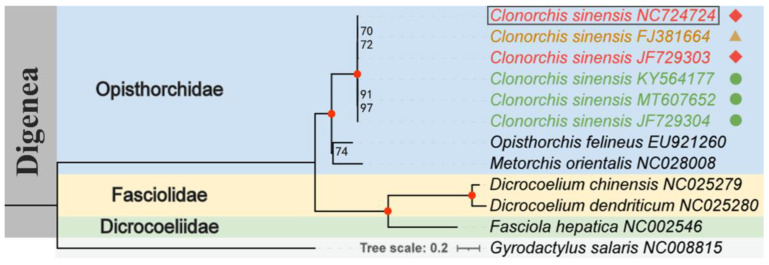
Phylogenetic relationships of *C. sinensis* (Cs-c2), other regional isolates of the species, and closely related digenean trematodes based on *ND5*, *ND6*, and *ND1* genes. A phylogenetic tree was constructed using the maximum likelihood (ML) algorithm. ML bootstrap support values are shown at each node; red dots indicate ML = 100, and other values are provided above the nodes. Isolates from China, Russia, and South Korea are marked with red (diamonds), brown (triangles), and green (circles), respectively. *Gyrodactylus salaris* (NC008815) was used as the outgroup.

For Dataset 3, ML analysis was used to construct a phylogenetic tree using concatenated nucleotide sequences of two rapidly evolving genes *(ND5* and *ND6*) and one slowly evolving gene (*ND1*) from intraspecific PCGs of *C. sinensis*, with *Gyrodactylus salaris* (GenBank accession number: NC008815.1) as the outgroup (Figure 3). Analysis of clustering patterns of *C. sinensis* from different regions revealed that strains from China, Russia, and South Korea clustered into two clades: Chinese and Russian strains exhibited a closer genetic relationship, whereas South Korean strains had diverged, even showing significant intraregional population differences. All key nodes in the phylogenetic tree provided reliable support, which is consistent with the explanation that minor mt genome sequence variations in the same species can be attributed to large geographical distances between sampling sites [23]. This study resolved the ambiguous regional clustering of *C. sinensis* in previous findings [21].

In the phylogenetic tree constructed from Dataset 3, all the trematodes in Opisthorchiidae formed a monophyletic group. *C. sinensis* was more closely related to *Opisthorchis felineus* than to *Metorchis orientalis*, with the three species forming sister groups, which is consistent with the findings of previous studies [46]. The phylogenetic tree constructed in this study also distinguished Opisthorchiidae from two other closely related families (Fasciolidae and Dicrocoeliidae), which is consistent with the findings of other similar studies. Furthermore, although molecular markers have revealed some geographical population differences, the lack of samples from a broad range of hosts may mask cryptic genetic lineages—variations that are easily overlooked in traditional small-sample studies [31]. Thus, integrating *C. sinensis* samples from multiple hosts (including wild hosts) and using whole-genome sequencing to analyse their genetic structure is expected not only to identify new genotypes or cryptic species but also to provide key molecular evidence for tracing transmission chains and designing targeted control measures (e.g., vaccine target screening). This research direction holds significant practical value for overcoming the current bottlenecks in control technologies [54].

**Figure 4:**
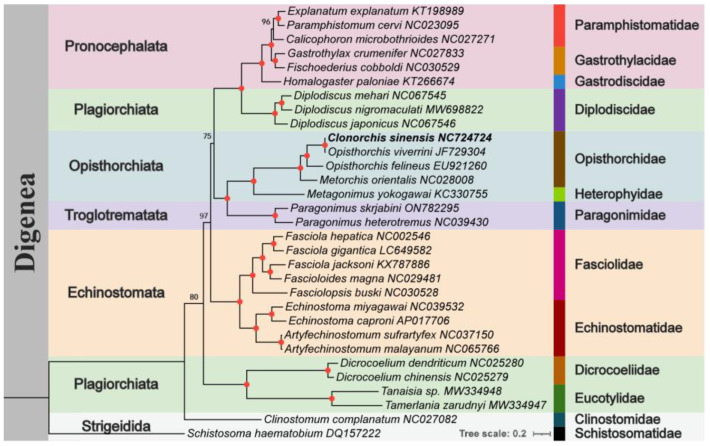
Phylogenetic relationships of *C. sinensis* (Cs-c2) and other selected digenean trematodes based on *ND4*, *ND5*, *ND1*, *ND2*, and *COX1* genes. A phylogenetic tree was constructed using the ML algorithm. ML bootstrap support values are shown at each node; red dots indicate ML = 100, and other values are provided above the nodes. Suborders and families within Digenea are highlighted in different colours. *Schistosoma haematobium* (DQ157222) was used as the outgroup.

For Dataset 4, a phylogenetic tree was constructed using ML analysis with concatenated nucleotide sequences of three rapidly evolving genes (*ND4*, *ND5*, *ND1*), one moderately evolving gene (*ND2*), and one slowly evolving gene (*COX1*). *Schistosoma haematobium* (Schistosomatidae, GenBank accession number: DQ157222) was used as the outgroup (Figure 4). The phylogenetic framework generated by the multimolecular marker strategy supported robust clustering of 13 families within Digenea, confirming the monophyly of all represented families and providing molecular systematic support for subordinal classifications. The topological structure of family positions and intrafamily branches was consistent with previous mt genome-based studies [17,55,56].

Notably, phylogenetic analysis supported the placement of Eucotylidae within the suborder Digenea, as it was closely related to Dicrocoeliidae [57], which is consistent with prior research [58]. However, our analysis did not support the assignment of Diplodiscidae to Plagiorchiata, indicating that the composition and interfamily relationships of Plagiorchiata require re-evaluation with more data. Furthermore, regardless of the dataset or model employed, the systematic characteristics of the suborders Echinostomata [58], Pronocephalata [59], and Opisthorchiata [60] examined in this study were partially corroborated by their congruence with previous conclusions based on rRNA genes [30]. However, the phylogeny of Plagiorchiata and Strigeidida needs further investigation with additional sequences.

Inconsistencies between nuclear gene-based and mt genome phylogenies are not uncommon [61], and trematode classification continues to be dynamically adjusted by molecular studies [62]. Taxonomic discrepancies exist, with different subordinal names used in the literature [63]. For example, the subordinal classification of Eucotylidae and Diplodiscidae remains controversial, as traditional taxonomy relies on morphological traits (e.g., sucker structure and reproductive organ morphology), whereas molecular systematics (e.g., based on 18S rRNA or the mt genome) reveal hidden evolutionary relationships, leading to subordinal reclassifications. Therefore, interfamily relationships within Digenea require more cautious re-evaluations using additional sequence data (e.g., combined analyses of mitochondrial and nuclear genes) and more precise sequencing methods [51].

## 4. Conclusions

In this study, we sequenced the complete mt genome of fish-borne *C. sinensis* from Changchun (Cs-c2) and reported the results of its analysis, thus addressing the gap in mitochondrial data concerning the Northeast Chinese geographical strain of *C. sinensis*. By calculating the π and Ka/Ks ratios and combining the phylogenetic results from single genes, we evaluated and screened the evolutionary rates and phylogenetic resolution of multiple mt-genome PCGs with different evolutionary speeds across taxonomic levels. The multimolecular marker identification strategy employed in this study, which is based on PCGs in the mt genome (i.e., constructing phylogenetic trees using concatenated nucleotide sequences of high-resolution genes with different evolutionary rates, including *ND5*, *ND6*, and *ND1*), yielded results sufficient to distinguish recent divergence events among *C. sinensis* strains from different geographical regions (China, South Korea, and Russia).

Using the same strategy, the ML phylogenetic tree constructed by concatenating *ND4*, *ND5*, *ND1*, *ND2*, and *COX1* supported the interrelationships among suborders and families within Digenea, as well as the phylogenetic affinities between the controversial suborder Plagiorchiata and the families Eucotylidae and Diplodiscidae [62]. This study also clarified the interspecific and intergeneric relationships among the suborders Echinostomata, Pronocephalata, and Opisthorchiata [59,60,61]. However, certain discrepancies between the phylogenetic relationships of Plagiorchiata and Strigeidida inferred herein and those reported in previous studies based on single molecular markers or morphological analyses remain [57]. Thus, the results of the present study may provide molecular evidence for the evolutionary history of six suborders and thirteen families within Digenea.

Analyses of Ka/Ks revealed that *ND5* and *ND6* are superior molecular markers than commonly used *COX1* and *ND1* for studying recent intraspecific divergence in *C. sinensis*. To resolve higher-order taxonomic divergence and population-level relationships within Digenea, *ND4*, *ND5*, and *ND1* may offer higher phylogenetic resolution than traditional “species barcodes” [46].

Moreover, considering that substitution saturation in high-mutation-rate genes and noncoding regions can significantly affect phylogenetic analyses at higher taxonomic levels [64], the inclusion of one or two slowly evolving genes helps stabilize deep nodes, thereby mitigating this issue to a certain extent. The use of PCG combinations not only reduces the computational data volume but also effectively minimizes the interference of genetic noise [65], ultimately improving the resolution of phylogenetic tree construction. The results of the concatenated multigene phylogenetic tree analysis in this study confirmed the validity of our hypotheses; however, the applicability of this strategy requires careful evaluation in future research.

## Data Availability

The original contributions presented in this study are included in the article/Supplementary Materials. Further inquiries can be directed to the corresponding authors.

## Supplementary Materials

The following supporting information can be downloaded at: https://www.mdpi.com/article/10.3390/biom15091246/s1, Figure S1: Gross and light microscopic observations; Figure S2: Secondary structures of Transfer RNA (tRNA) families in *C. sinensis* mtDNA; Figure S3: Secondary structures of ribosomal RNA (rRNA) families in *C. sinensis* mtDNA; Figure S4: mtDNA Codon families are labeled on the *x*-axis; Figure S5: Maximum likelihood (ML) phylogenetic trees constructed from each of the 12 PCGs in mt genomes of *C. sinensis* (Cs-c2), other regional isolates of the species, and close-related digenean trematodes; Figure S6: Maximum likelihood (ML) phylogenetic trees constructed from individual PCGs of *C. sinensis* (Cs-c2) and other selected digenean trematodes from NCBI; Table S1: Primers used for amplifying the complete mitochondrial genome of *C. sinensis*; Table S2: Base Composition of *C. sinensis* Mitochondria; Table S3: Best-fit models for all maximum likelihood (ML) trees constructed based on PCGs in Datasets 3 and 4 of this study; Table S4: Annotations of the mtDNA of *C. sinensis*; Table S5: Mt genomes of *C. sinensis* from different geographical origins.

## Abbreviations

PCGs:	Protein-coding genes
Mt:	Mitochondrial
R2DT:	RNA 2D Structure
Ka/Ks:	Non-synonymous/synonymous mutation ratio
RSCU:	Relative synonymous codon usage
tRNA:	Transfer RNA
rRNA:	Ribosomal RNA
Pi (π):	Nucleotide diversity
ML:	Maximum likelihood
