# Supplementary material for: A Novel Clonorchis sinensis Mitogenome: Elucidating Multiregional Strain Phylogeny and Revising the Digenean Mitochondrial Genome Tree
Source: Biomolecules. 2025 Aug 28;15(9):1246. doi: 10.3390/biom15091246 (PMC12467321; doi:10.3390/biom15091246)
Supplement: Supplementary file 1 [file biomolecules-15-01246-s001.zip › biomolecules-3766470-supplementary.pdf]

SUPPLIMENTARY DATA

Additional File 1: Figure S1. Gross and light microscopic observations.

A. Infected fish specimens collected in the experiment.

B. Encysted metacercariae collected by artificial digestion method, observed under light microscope (×40 magnification).

C. Adult worms successfully isolated from the animal model.

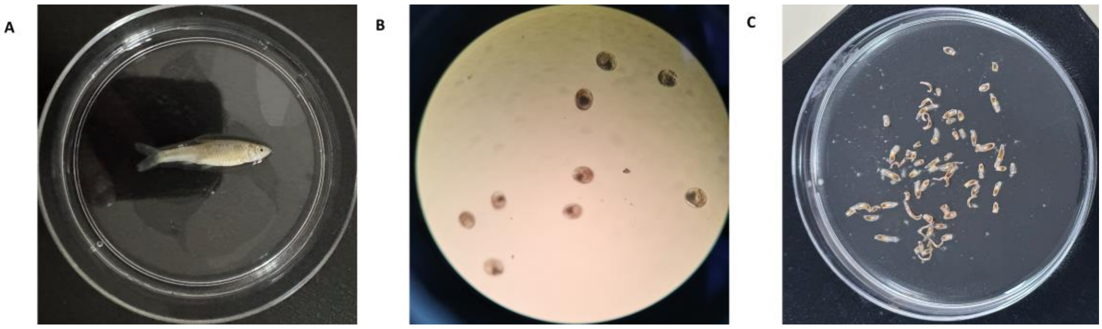

Additional File 2: Table S1 Primers used for amplifying the complete mitochondrial genome of *C. sinensis.*

Name of primer	Sequence (5' to 3')
CSNAD5R	CGCACCTGGGTGGGTAGTAAACA
CSNAD5F	ATGAGTTGATTACCGTTTCARGCGGCGTG
CSNAD4R	ACCACCGCAACAAAGAYCTCATAAAAAAACA
CSNAD4F	GATAGTACATGCTGAGGCCAGCA
CSNAD1R	AACCGATCCCAACCAAGAAAAAAT
CSNAD1F	TCGGTTGGTTGGGTGTGTTTTTATTT
CSCOX1R	CCAGGGTCTCTAATCCTCTTCTTTCTAT
CSCOX1F	CCCAAATATAATAAGTAAGACGGAAAG

Additional File 3: Figure S2 Secondary structures of Transfer RNA(tRNA) families in *C. sinensis* mtDNA.

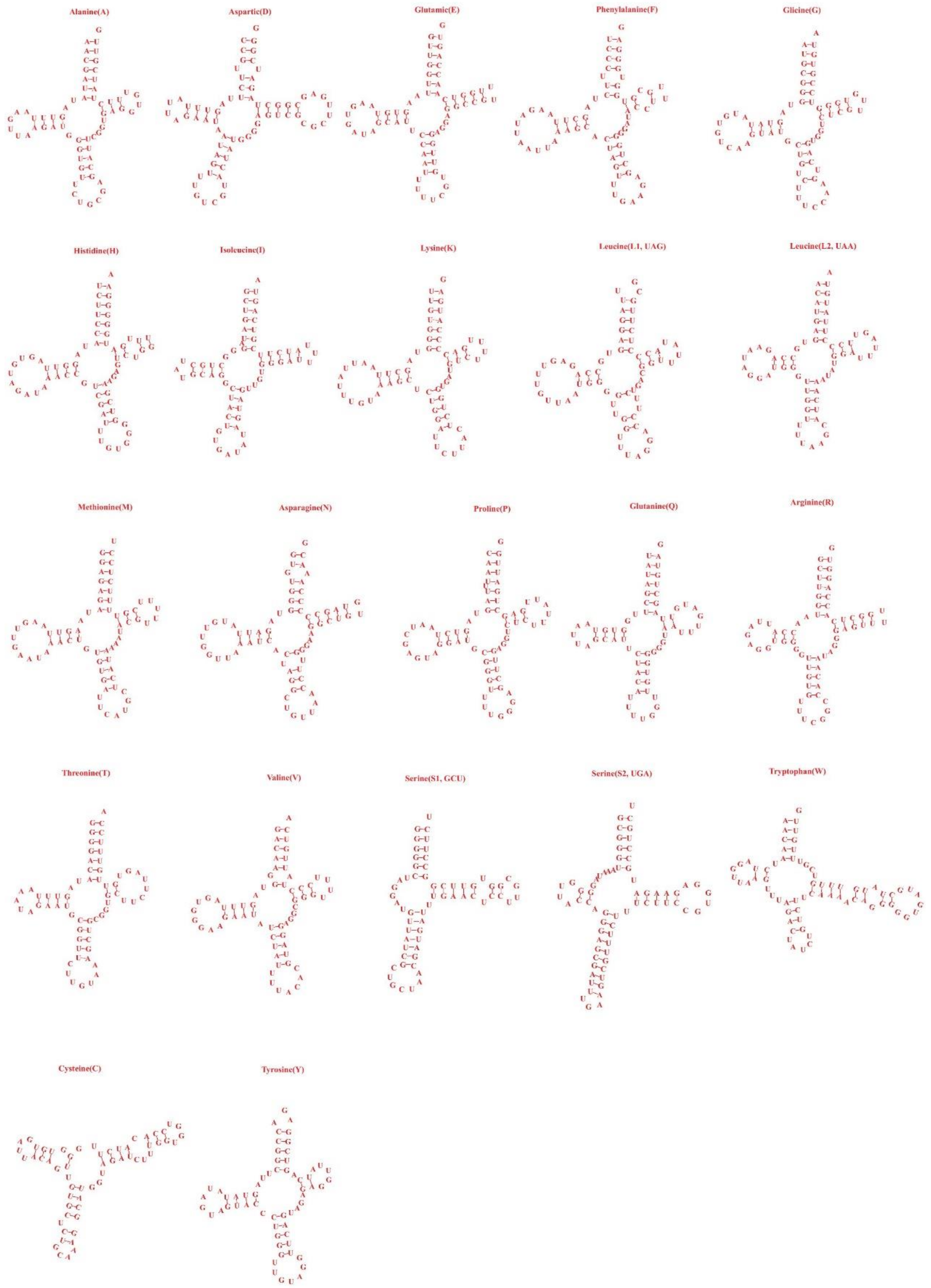

Additional File 4: Figure S3 Secondary structures of ribosomal RNA（rRNA） families in *C. sinensis* mtDNA. *rrnL* (left) and *rrnS* (right).

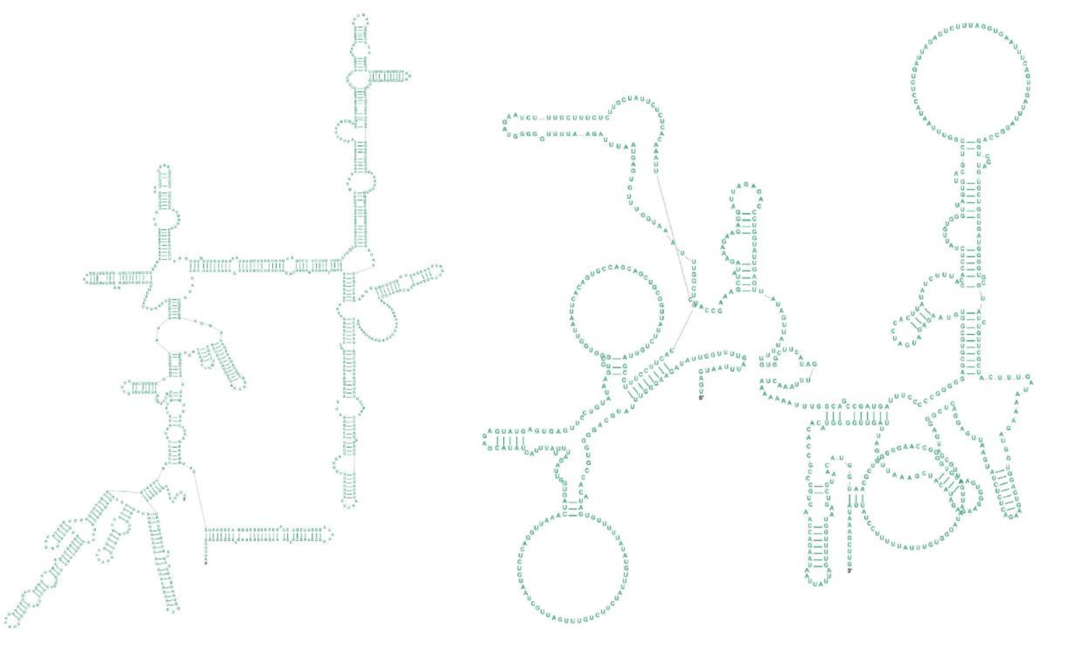

Additional File 5: Table S2 Base Composition of *C. sinensis* Mitochondria. Table lists the nucleotide composition and skewness of major components of *C. sinensis* mtDNA, control region (CR), and each codon position (1, 2, 3).

**Item**	**Size**	**Nucleotide composition（%）**					**AT-Skew**	**GC-Skew**
-	bp	A	G	T	C	AT	Skew	Skew
All gene	13410	17.25	27.54	42.81	12.4	60.06	-0.43	0.38
PCGs	10185	15.67	27.35	45.08	11.9	60.75	-0.48	0.39
1st codon position	3395	18.5	29.96	38.14	13.4	56.64	-0.35	0.38
2st codon position	3395	15.7	21.94	47.66	14.7	63.36	-0.50	0.20
3st codon position	3395	12.81	30.16	49.43	7.6	62.24	-0.59	0.60
12S RNA（rrnS）	779	24.65	25.67	35.3	14.38	59.95	-0.18	0.28
16S RNA（rrnL）	999	21.22	28.23	37.84	12.71	59.06	-0.28	0.38
tRNA	1447	21.63	29.37	34.35	14.65	55.98	-0.23	0.33
CR	412	25.49	25.97	35.19	13.11	60.68	-0.16	0.33

Note: AT-Skew = [A−T]/[A+T]; GC-Skew = [G−C]/[G+C].

Additional File 6 Figure S4: mtDNA Codon families are labeled on the x-axis. The segments of color-coded bars on the y-axis represent the proportion of each codon used to encode the corresponding amino acid.

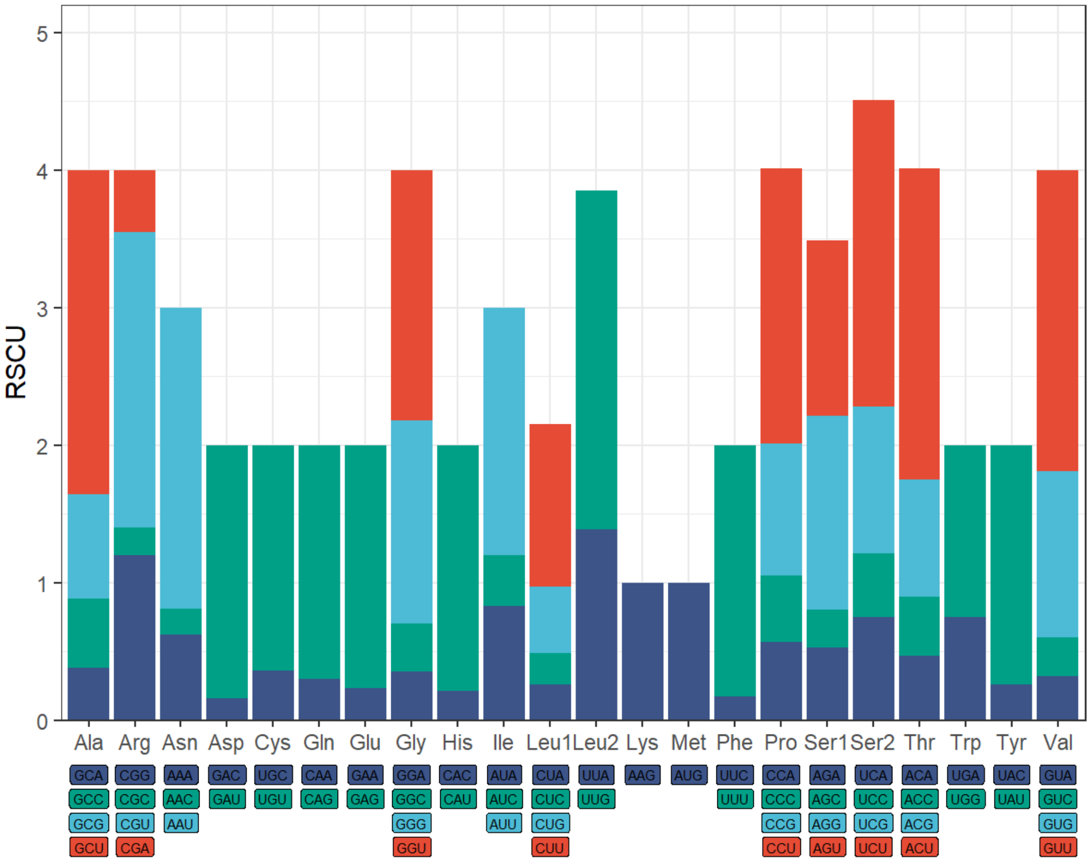

Additional File 7: Figure S5. Maximum likelihood (ML) phylogenetic trees constructed from each of the 12 protein-coding genes (PCGs) in mtDNAs of *C. sinensis* (Cs-c2), other regional isolates of the species, and close-related digenean trematodes. PCGs finally selected for concatenated phylogenetic tree construction are marked with red asterisks. ML Bootstrap support values are shown above the nodes. Bootstrap values at each node and clustering patterns of *C. sinensis* from different regions are depicted. Isolates from China, Russia, and South Korea are marked with red (diamonds), brown (triangles), and green (circles), respectively. *Gyrodactylus salaris* (NC008815) was used as the outgroup.

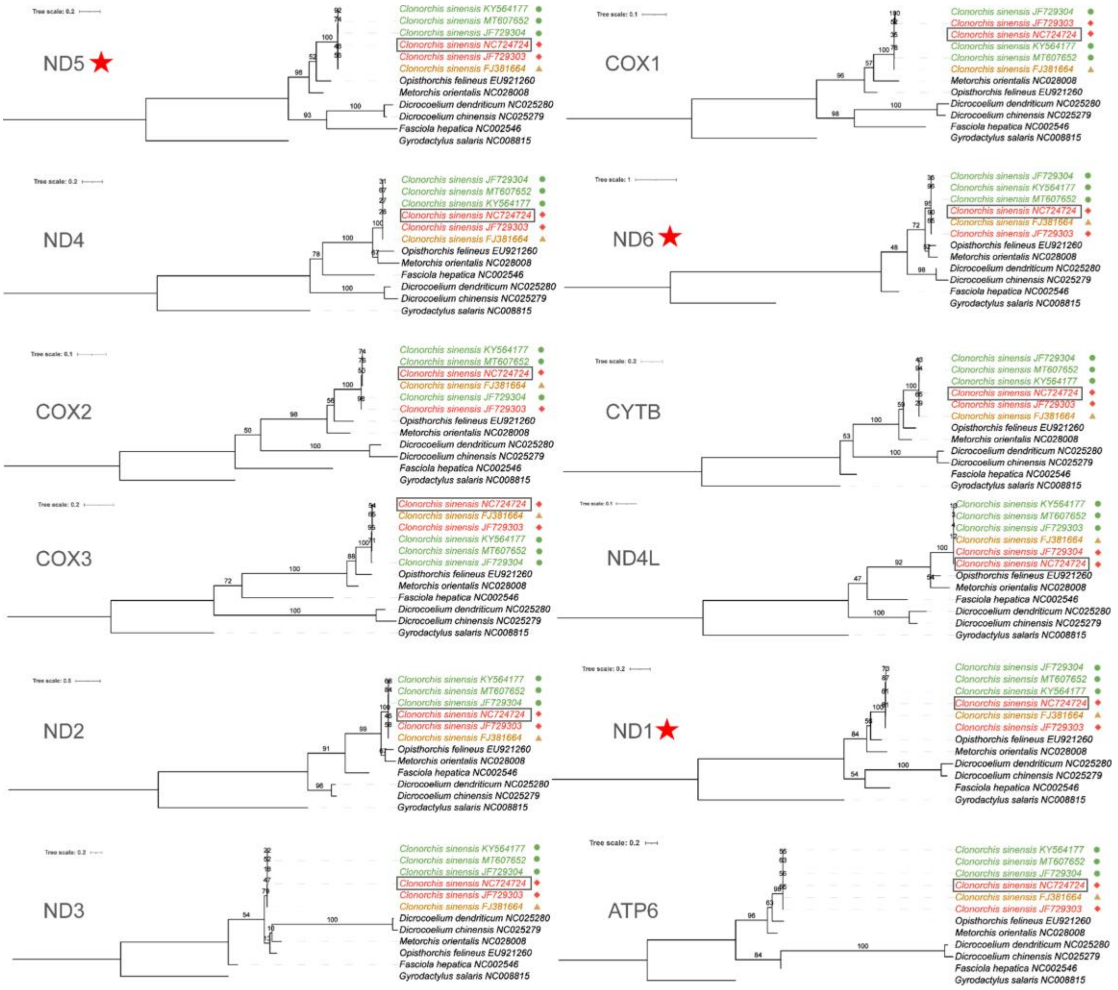

Additional File 8: Figure S6. Maximum likelihood (ML) phylogenetic trees constructed from individual protein-coding genes (PCGs) of *C. sinensis* (Cs-c2) and other selected digenean trematodes from NCBI. PCGs finally selected for concatenated phylogenetic tree construction in the manuscript are marked with red asterisks. Approximate ML Bootstrap support values are visually represented by different colors in the figure. Clustering patterns of digenean trematodes in the ML trees are shown. *Schistosoma haematobium* (DQ157222) was used as the outgroup.

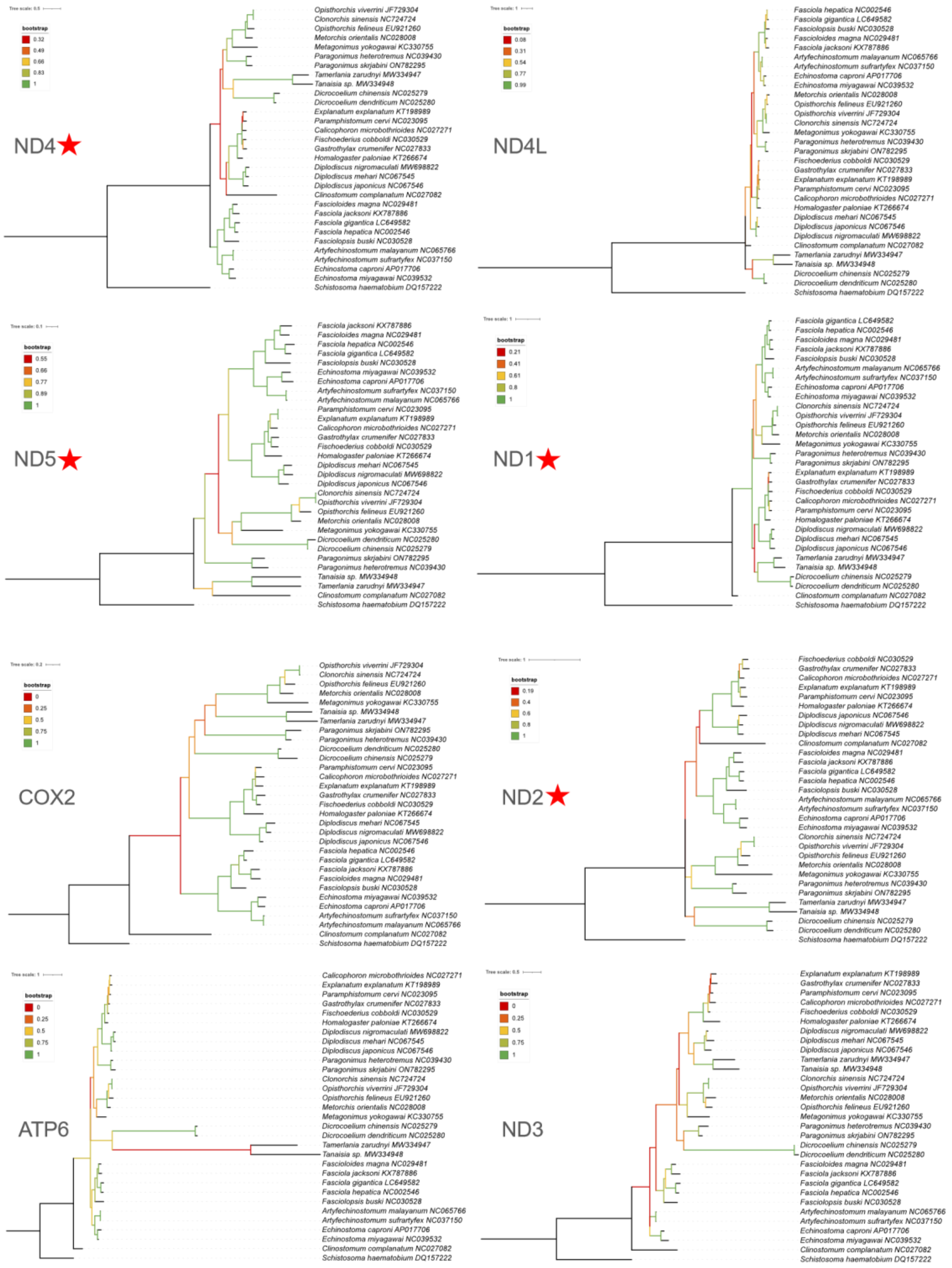

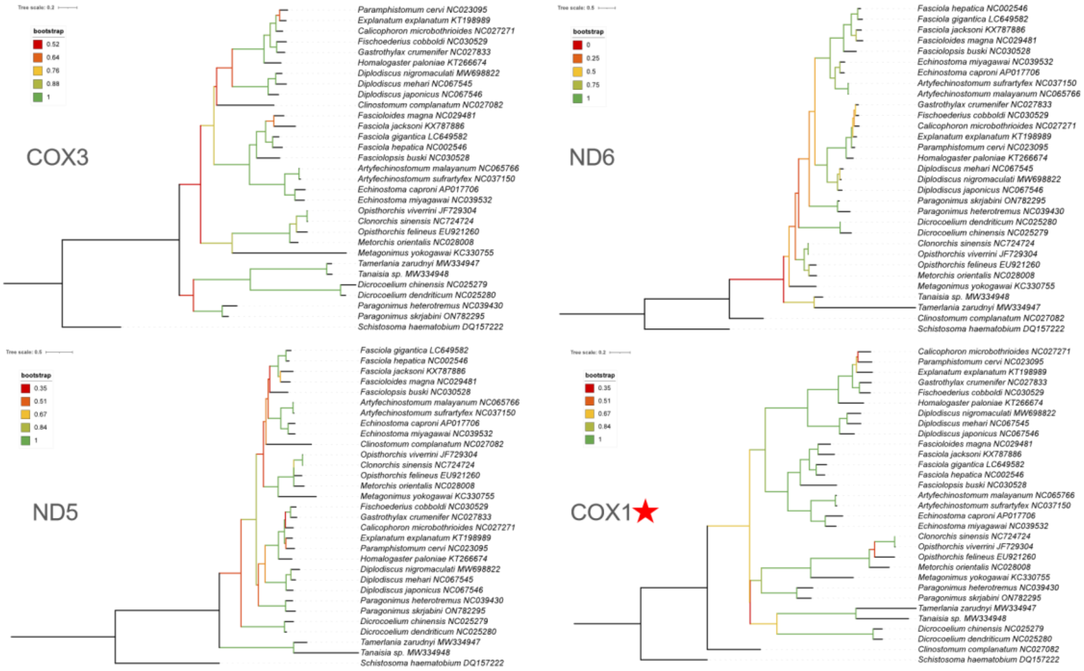

Tree scale: 0.2
bootstrap
0.52
0.64
0.76
0.88
1
COX3
Paramphistomum cervi NC023095
Explanatum explanatum KT198989
Calicophoron microbothrioides NC027271
Fischoederius cobboldi NC030529
Gastrothylax crumenifer NC027833
Homalogaster paloniae KT266674
Diplodiscus nigromaculati MW698822
Diplodiscus mehari NC067545
Diplodiscus japonicus NC067546
Clinostomum complanatum NC027082
Fascioloides magna NC029481
Fasciola jacksoni KX787886
Fasciola gigantica LC649582
Fasciola hepatica NC002546
Fasciolopsis buski NC030528
Artyfechinostomum malayanum NC065766
Artyfechinostomum sufrartyfex NC037150
Echinostoma caproni AP017706
Echinostoma miyagawai NC039532
Opisthorchis viverrini JF729304
Clonorchis sinensis NC724724
Opisthorchis felineus EU921260
Metorchis orientalis NC028008
Metagonimus yokogawai KC330755
Tamerlania zarudnyi MW334947
Tanaisia sp. MW334948
Dicrocoelium chinensis NC025279
Dicrocoelium dendriticum NC025280
Paragonimus heterotremus NC039430
Paragonimus skrjabini ON782295
Schistosoma haematobium DQ157222
Tree scale: 0.5
bootstrap
0
0.25
0.5
0.75
1
ND6
Fasciola hepatica NC002546
Fasciola gigantica LC649582
Fasciola jacksoni KX787886
Fascioloides magna NC029481
Fasciolopsis buski NC030528
Echinostoma miyagawai NC039532
Echinostoma caproni AP017706
Artyfechinostomum sufrartyfex NC037150
Artyfechinostomum malayanum NC065766
Gastrothylax crumenifer NC027833
Fischoederius cobboldi NC030529
Calicophoron microbothrioides NC027271
Explanatum explanatum KT198989
Paramphistomum cervi NC023095
Homalogaster paloniae KT266674
Diplodiscus mehari NC067545
Diplodiscus nigromaculati MW698822
Diplodiscus japonicus NC067546
Paragonimus skrjabini ON782295
Paragonimus heterotremus NC039430
Dicrocoelium dendriticum NC025280
Dicrocoelium chinensis NC025279
Clonorchis sinensis NC724724
Opisthorchis viverrini JF729304
Opisthorchis felineus EU921260
Metorchis orientalis NC028008
Metagonimus yokogawai KC330755
Tanaisia sp. MW334948
Tamerlania zarudnyi MW334947
Clinostomum complanatum NC027082
Schistosoma haematobium DQ157222
Tree scale: 0.5
bootstrap
0.35
0.51
0.67
0.84
1
ND5
Fasciola gigantica LC649582
Fasciola hepatica NC002546
Fasciola jacksoni KX787886
Fascioloides magna NC029481
Fasciolopsis buski NC030528
Artyfechinostomum malayanum NC065766
Artyfechinostomum sufrartyfex NC037150
Echinostoma caproni AP017706
Echinostoma miyagawai NC039532
Clinostomum complanatum NC027082
Opisthorchis viverrini JF729304
Clonorchis sinensis NC724724
Opisthorchis felineus EU921260
Metorchis orientalis NC028008
Metagonimus yokogawai KC330755
Fischoederius cobboldi NC030529
Gastrothylax crumenifer NC027833
Calicophoron microbothrioides NC027271
Explanatum explanatum KT198989
Paramphistomum cervi NC023095
Homalogaster paloniae KT266674
Diplodiscus nigromaculati MW698822
Diplodiscus mehari NC067545
Diplodiscus japonicus NC067546
Paragonimus heterotremus NC039430
Paragonimus skrjabini ON782295
Dicrocoelium chinensis NC025279
Dicrocoelium dendriticum NC025280
Tamerlania zarudnyi MW334947
Tanaisia sp. MW334948
Schistosoma haematobium DQ157222
Tree scale: 0.2
bootstrap
0.35
0.51
0.67
0.84
1
COX1★
Calicophoron microbothrioides NC027271
Paramphistomum cervi NC023095
Explanatum explanatum KT198989
Gastrothylax crumenifer NC027833
Fischoederius cobboldi NC030529
Homalogaster paloniae KT266674
Diplodiscus nigromaculati MW698822
Diplodiscus mehari NC067545
Diplodiscus japonicus NC067546
Fascioloides magna NC029481
Fasciola jacksoni KX787886
Fasciola gigantica LC649582
Fasciola hepatica NC002546
Fasciolopsis buski NC030528
Artyfechinostomum malayanum NC065766
Artyfechinostomum sufrartyfex NC037150
Echinostoma caproni AP017706
Echinostoma miyagawai NC039532
Clonorchis sinensis NC724724
Opisthorchis viverrini JF729304
Opisthorchis felineus EU921260
Metorchis orientalis NC028008
Metagonimus yokogawai KC330755
Paragonimus heterotremus NC039430
Paragonimus skrjabini ON782295
Tamerlania zarudnyi MW334947
Tanaisia sp. MW334948
Dicrocoelium chinensis NC025279
Dicrocoelium dendriticum NC025280
Clinostomum complanatum NC027082
Schistosoma haematobium DQ157222

Additional File 9: Table S3. Best-fit models for all maximum likelihood (ML) trees constructed based on protein-coding genes (PCGs) in Datasets 3 and 4 of this study.

**PCGs**	**Best-fit models**	**Datasets （1'/2'3/4）**
ATP6	TN93+G+I	1'
COX1	HKY+G	1'
COX2	HKY+G	1'
COX3	HKY+G+I	1'
CYTB	TN93+G	1'
ND1	HKY+G	1'
ND2	TN93+G+I	1'
ND3	HKY+G	1'
ND4	TN93+G+I	1'
ND4L	HKY+G+I	1'
ND5	TN93+G+I	1'
ND6	TN93+G	1'
ATP6	GTR+G	2'
COX1	GTR+G+I	2'
COX2	GTR+G+I	2'
COX3	GTR+G+I	2'
CYTB	GTR+G+I	2'
ND1	GTR+G+I	2'
ND2	GTR+G+I	2'
ND3	GTR+G	2'
ND4	GTR+G+I	2'
ND4L	HKY+G	2'
ND5	GTR+G+I	2'
ND6	TN93+G	2'
ND5+ND6+ND1	TN93+G+I	3
ND4+ND5+ND1+COX1+ATP6	GTR+G+I	4

Additional File 10: Table S4. Annotations of the mtDNA of *C. sinensis*.

Gene	Location	Size(bp)	AA	Start/Stop codon	Anticodon	Spacer (+)/ Overlap (-)
COX3	1-642	642	213	ATG/TAG		30
tRNA - His (H)	673-739	67			GTG	8
CYTB	748-1860	1113	370	ATG/TAG		8
ND4L	1869-2132	264	87	ATG/TAG		-40
ND4	2093-3370	1278	425	GTG/TAG		12
tRNA - Gln (Q)	3383-3445	63			TTG	17
tRNA - Phe (F)	3463-3530	68			GAA	-1
tRNA - Met (M)	3530-3597	68			CAT	0
ATP6	3598-4113	516	171	ATG/TAG		35
ND2	4149-5021	873	290	GTG/TAG		8
tRNA - Val (V)	5030-5094	65			TAC	17
tRNA - Ala (A)	5112-5174	63			TGC	5
tRNA - Asp (D)	5180-5248	69			GTC	3
ND1	5252-6154	903	300	GTG/TAG		-1
tRNA - Asn (N)	6154-6222	69				7
tRNA - Pro (P)	6230-6298	69			GTT	-1
tRNA - Ile (I)	6298-6361	64			TGG	21
tRNA - Lys (K)	6383-6450	68			GAT	3
ND3	6454-6810	357	118	GTG/TAG	CTT	10
tRNA - Ser^{AGN} (S1)	6821-6881	61			GCT	17
tRNA - Trp (W)	6899-6967	69			TCA	27
COX1	6995-8530	1536	519	GTG/TAA		13
tRNA - Thr (T)	8544-8607	64			TGT	0
16S RNA（rrnL）	8608-9606	999				0
tRNA - Cys (C)	9607-9665	59			GCA	0
12S RNA（rrnS）	9666-10444	779				0
COX2	10445-11080	636	211	ATG/TAG		19
ND6	11100-11561	462	153	GTG/TAA		6
tRNA - Tyr (Y)	11568-11629	62			GTA	0
tRNA - Leu^{CUN} (L1)	11630-11695	66			TAG	-3
tRNA - Ser^{UCN} (S2)	11693-11761	69			TGA	4
tRNA - Leu^{UUR} (L2)	11766-11830	65			TAA	12
tRNA - Arg (R)	11843-11907	65			TCG	1
ND5	11909-13513	1605	534	GTG/TAA		10
tRNA - Glu (E)	13524-13590	67			TTC	0
Long Non - coding region (LNCR)	13591-13744	153				
CR*	13591-14002	412				
tRNA - Gly (G)	14003-14069	67			TCC	412
Short Non - coding region (SNCR)	14070-14136	67				

Additional File 11: Table S5 Mt genomes of *C. sinensis* from different geographical origins.

Species (strain)	Geographic origin	GenBank accession no.	Identity (vs Cs-c2)	References
C. sinensis (Cs-c2)	China	NC724724	-	Present study
C. sinensis (Cs-r1)	Russia	FJ381664	99.88%	Shekhovtsov et al. [47]
C. sinensis	Korea	MT607652	99.76%	Kinkar et al. [11]
C. sinensis (Cs-k2)	Korea	KY564177	99.74%	D. Wang et al. [21]
C. sinensis (Cs-c1)	China	JF729303	99.68%	Cai et al. [46]
C. sinensis (Cs-k1)	Korea	JF729304	99.60%	Cai et al. [46]
